# Author's response to comments on "Epidemiologic Measures and Policy Formulation"

**DOI:** 10.1186/1742-7622-2-2

**Published:** 2005-05-19

**Authors:** Sander Greenland

**Affiliations:** 1 Departments of Epidemiology and Statistics, University of California, Los Angeles, USA

I see no important disagreement between me and the commentators, but disagreement does exist on these topics. I would classify opposing views into two major categories. On one side, some writers assert that causal inference can and should be done without counterfactuals. In my view, Dawid [1,2] is a moderate on that side, as he deploys devices that are isomorphic to counterfactuals, so the difference seems mostly one of labeling and emphasis (which is not always unimportant). More radical counterfactual deniers include Shafer [3], who appears mostly upset because counterfactual models continue to weave into the foundation of statistics, econometrics, sociology, and health sciences, while his approach [4] appears destined for the dustbin, along with other truly noncounterfactual theories of causation. Pearl [5] gives a succinct account of the failings of these theories (reference 5, section 7.5), noting (as do Greenland and Brumback [6]) that the causal models entering into scientific teaching and application (including causal graphs, causal "pies," and structural equations) have mappings into counterfactual formalisms.

The target of my article was the other side, those who take counterfactuals uncritically or superficially, without paying enough attention to what these hypothetical quantities are supposed to mean. My article originated as a chapter in a WHO volume [7]. This volume arose from a conference which seemed a festival of counterfactual abuse, rife with talk of cause-of-death removal as if it were an intervention. It is disheartening if not frightening to witness discussion of global health policy framed in such terms. In this context, the concerns expressed by Dawid [1,2] about counterfactuals seem reserved.

Counterfactual abuse can be diminished by connecting potential outcomes to interventions. Susser and Schwartz [8] point out that this connection is needed for lifestyle risk factors (smoking, physical inactivity, etc.) just as for social factors. I agree; risk-factor epidemiology could better serve public health if it addressed what could be done, rather than estimating effects of the unattainable (like removal of all tobacco exposure), without regard to how change is brought about.

Becoming more realistic involves more than just operationalizing the exposure (right-hand) side of the structural equation; one also needs to expand the left-hand side to consider the full spectrum of intervention effects, such as all effects of smoking cessation (e.g., weight gain, depression). The traditional narrow focus on a few prominent endpoints (like cancer and cardiovascular disease), encouraged by the case-control viewpoint, has discouraged grappling with the multivariate complexity of outcomes as well as exposures. Worse, in the smoking context, there may be a bias against acknowledging that one of our most damaging population exposures (tobacco) may bring worthwhile benefits to a non-negligible portion of the population – not just medical benefits like Parkinsonism prevention, but also psychologic benefits like enhanced sense of well-being, which are hard to measure and weigh against costs. If we really believe in informed consent, then we must inform the public about how lifestyle choices are not just about lifespan maximization, but are also choices of how to live and die. This view will not sit well with those for whom good sensations are evil if the sensations do not come from sanctified sources like religious faith, licensed entertainment, or prescription drugs.
